# Post-Fire Spatial Patterns of Soil Nitrogen Mineralization and Microbial Abundance

**DOI:** 10.1371/journal.pone.0050597

**Published:** 2012-11-30

**Authors:** Erica A. H. Smithwick, Kusum J. Naithani, Teri C. Balser, William H. Romme, Monica G. Turner

**Affiliations:** 1 Department of Geography and Intercollege Graduate Degree Program in Ecology, The Pennsylvania State University, University Park, Pennsylvania, United States of America; 2 University of Florida, Department of Soil and Water Science, College of Agricultural and Life Sciences, Gainesville, Florida, United States of America; 3 Graduate Degree Program in Ecology, Colorado State University, Fort Collins, Colorado, United States of America; 4 Department of Zoology, University of Wisconsin, Madison, Wisconsin, United States of America; Argonne National Laboratory, United States of America

## Abstract

Stand-replacing fires influence soil nitrogen availability and microbial community composition, which may in turn mediate post-fire successional dynamics and nutrient cycling. However, fires create patchiness at both local and landscape scales and do not result in consistent patterns of ecological dynamics. The objectives of this study were to (1) quantify the spatial structure of microbial communities in forest stands recently affected by stand-replacing fire and (2) determine whether microbial variables aid predictions of *in situ* net nitrogen mineralization rates in recently burned stands. The study was conducted in lodgepole pine (*Pinus contorta* var. *latifolia*) and Engelmann spruce/subalpine fir (*Picea engelmannii*/*Abies lasiocarpa*) forest stands that burned during summer 2000 in Greater Yellowstone (Wyoming, USA). Using a fully probabilistic spatial process model and Bayesian kriging, the spatial structure of microbial lipid abundance and fungi-to-bacteria ratios were found to be spatially structured within plots two years following fire (for most plots, autocorrelation range varied from 1.5 to 10.5 m). Congruence of spatial patterns among microbial variables, *in situ* net N mineralization, and cover variables was evident. Stepwise regression resulted in significant models of *in situ* net N mineralization and included variables describing fungal and bacterial abundance, although explained variance was low (R^2^<0.29). Unraveling complex spatial patterns of nutrient cycling and the biotic factors that regulate it remains challenging but is critical for explaining post-fire ecosystem function, especially in Greater Yellowstone, which is projected to experience increased fire frequencies by mid 21^st^ Century.

## Introduction

Fire is a complex spatial event that both responds to and produces heterogeneity in ecological systems [1], [2]. This complexity is characterized by variation in fire size, severity, frequency, and the location and timing of ignition factors [3], [4]. In turn, these complexities in fire regimes lead to spatial and temporal variability in post-fire effects such as gaseous emissions, vegetation dynamics, and biogeochemical cycling [5], [6], [7]. Complexity in fire patterns has been described at global and landscape scales [8], [9]. Yet, despite increased understanding of heterogeneity at broad scales, there is a paucity of studies that explore fine-scale spatial patterning in post-fire environments.

Fires modify the distribution and mass of nutrient elements in ecosystems through pyrolysis (thermal decomposition of organic matter by fire), volatilization (gaseous loss through combustion), and ash deposition [10]. As a result, fires modify nutrient availability [11], carbon storage [12], and ecosystem productivity [13]. Post-fire nutrient cycling also is modified by shifts in abiotic conditions, such as changes in soil moisture [14], temperature [15], pH [16], biotic substrate quality and quantity [17], and charcoal deposition [18]. Agents of these biogeochemical transformations in post-fire environments are the soil microbial organisms that are active following fire. In the absence of disturbance, soil microbial communities are spatially patterned at multiple, often hierarchical scales [19], [20], [21]. At fine scales (e.g., <1 to ∼10 m), microbial communities may vary in response to soil and root structure, plant species, litter inputs, nutrient cycling [22], and local microbial population dynamics [20], [23], while at broad scales (e.g., >10 m) patterns may reflect gradients in topography, vegetation type, land use, or soil [24], [25], [26]. Following disturbances such as fire, microbial abundance and community composition can be changed because of disruption of microbial microhabitats and altered resource availability [17], [27], [28]. Studies in agricultural or fertilized systems have reported changes in spatial variation and community structure [29], some of which may persist for decades [30]. However, despite repeated calls for increased understanding of ‘microbial biogeography’ [31], the scale(s) at which microbial communities are structured is unknown [32] and remains a key priority for understanding the ecological consequences of disturbance such as fire [33].

To characterize the role of microbial communities in these post-fire locations, we asked: what is the spatial structure of microbial communities in forest stands recently affected by stand-replacing fire? Spatial structuring of microbial lipids could be present in post-fire stands because of patchy microbial mortality caused by the fire event, or patchiness in post-fire soil resources; alternatively, homogenization of microbial communities could be expected in post-fire stands if fire reduced coupling between aboveground cover and soil N [34]. To explore this question, we used model-based geostatistics in a hierarchical Bayesian framework [35] to explore the spatial structure of microbial communities. A variety of classical kriging methods exists, including simple kriging which assumes a known constant spatial trend underlying the data, ordinary kriging which assumes an unknown constant trend, and universal kriging which assumes a general linear trend model [35]. In contrast, Bayesian kriging allows uncertainty in the model parameters to be reflected in the widths of the prediction intervals, thus providing a more reliable and realistic prediction of the spatial parameters of interest than traditional kriging methods [36]. Bayesian kriging also removes underlying spatial trends from the data prior to calculating range distances, allowing for more conservative inferences of spatial autocorrelation. Finally, by integrating observed data into spatial models, this approach allows for the estimation of ranges below the sampling grain (i.e., minimum distance between samples, 2 m), where traditional methods would detect insignificant spatial structure.

In addition to quantification of post-fire spatial structure of microbial communities, we asked: does microbial community information improve predictive models of soil N transformation? Given that microbes regulate nutrient mineralization, we expected that microbial community information could explain patterns of *in situ* net N mineralization rates [17]. The influence of microbial communities on post-fire N cycling is relevant in northern coniferous forests in the Rocky Mountains, where stand-replacing fires dominate the natural disturbance regime [37] and where ecosystem productivity appears to be limited by soil N availability [38]. Large fires are expected to become more common for these subalpine forests as a result of increased climate warming and drying [39].

This study builds upon an earlier study by Turner et al. [34] that analyzed fine-scale (2–20 m) spatial patterns of *in situ* net N mineralization, N pools, and aboveground cover during the first 4 years following fire in the Greater Yellowstone Ecosystem (GYE, Wyoming, USA). In this study, Turner et al. [34] used semivariograms to explore autocorrelation scales between N cycling and the cover of bare mineral soil, charred litter, live vegetation, and fresh litter. Results indicated little congruence in spatial range scales between soil N and aboveground cover, although coupling of aboveground cover and soil N was apparent at the individual sample point. Previously, Turner et al. [40] reported elevated rates of ammonium (NH_4_^+^) immobilization two years following fire, and Smithwick et al. [41] concluded that microbial community structure best explained patterns of gross ammonium (NH_4_^+^) mineralization across 20 mature forest stands in the GYE. These latter studies indicate a strong role of microbial dynamics at landscape scales in governing N dynamics. But because microbial community composition was not included in the fine-scale study by Turner et al. [34], it is unknown whether this information aids prediction of post-fire N cycling at fine spatial scales during the immediate post-fire years.

Understanding the influence of microbial communities on post-fire N cycling may shed light on patterns of post-fire vegetation recovery and further elucidate the relationship between above- and below-ground function in disturbed environments. This study therefore reports on new microbial data collected as part of the study described by [34]. We use new statistical approaches that rely on probabilistic spatial models (rather than semivariograms) to calculate range distances for *in situ* net N mineralization, cover, soil, and microbial variables. We also include the microbial information in predictive models of N mineralization, using N values reported by Turner et al. [34], to improve understanding of autocorrelation scales of microbial communities and N cycling two years following stand-replacing fire. Finally, we report on rates of laboratory isotopic pool dilution at two of the plots described by Turner et al. [34] in order to corroborate plot-level patterns in mineralization and microbial consumption following crown fire.

## Methods

Four plots previously described by Turner et al. [34] were selected for intensive microbial analysis. The plots represented two of the dominant forest types in the GYE (lodgepole pine (*Pinus contorta* var. *latifolia*) and Engelmann spruce/subalpine fir (*Picea engelmannii* Parry/*Abies lasiocarpa* Hook. Nutt.)), and two severities of stand-replacing fire (canopy vs. surface). Two of the plots were centered on the 1280-ha Glade Fire (hereafter referred to as Glade, 44° 5′ 18.936′′ N, 110° 43′ 23.931′′ W), which burned a mosaic of 120 and 150-yr old forest dominated by lodgepole pine that had developed after fires in 1879 or 1856 [42]. Elevation is approximately 2150 m and soils are derived from very infertile rhyolite substrates. Another two plots were centered on the 840-ha Moran fire (hereafter referred to as Moran, 43° 52′ 33.191′′ N, 110° 43′ 26.361′′ W) on the western shore of Jackson Lake. Prior to this fire, the plots were dominated by Engelmann spruce and subalpine fir (although lodgepole pine was locally present) and had not burned for >200 yrs. The substrate at Moran consists of glacial moraine deposits, including Precambrian crystalline and Paleozoic sedimentary rocks. Soils are characterized as Typic Chryocrepts. All four plots (Glade-crown, Glade-surface, Moran-crown, Moran-surface) experienced stand-replacing fire in late summer 2000 with 100% tree crown mortality and almost 100% consumption of the soil O horizon but were either crown fires, in which needles were consumed during the fire, or severe-surface fires, in which needles were killed and fell to the forest floor following the fire. Two years following the fire, during summer 2002, a 50 m×50 m intensive sampling grid [34] was used in each plot to sample fine-scale spatial variability of microbial communities. Sampling locations were spaced 2 m, 4 m, or 8 m apart along one of 9 rows, which were separated by 2 m. Each row had 9 cores, for a total of 81 soil cores per grid (plot). The sampling design was reversed in the middle three rows to account for anisotropy. This sampling design facilitated the study of spatial patterning by creating comparable power at different lag distances and maximizing sampling efficiency.

At each of 81 sampling locations per plot, clean PVC cores (5 cm radius×15 cm long) were used to collect soil samples for microbial analysis. Samples were kept cool in the field and shipped overnight to the University of Wisconsin (Madison, WI), where they were frozen for microbial analysis or stored at constant temperature prior to pool dilution analysis. Microbial lipid analysis (extraction of signature lipid biomarkers from the cell membrane and wall of microorganisms [43]) was used to assess the microbial community composition at each sampling location. Microbial lipid analysis is used for characterizing microbial communities because of its ability to capture a unique microbial ‘fingerprint’, estimate microbial biomass, and isolate microbial ‘biomarkers’ [21], [44]. The method is based on extraction and purification of phospholipid fatty acids (PLFA) from microbial cell membranes. Specific chemical and analytical methods have been described previously [41], [45]. Cores that did not have lipid 16∶0 present were deleted from the analysis (9 cores) and only lipids with chain lengths <20 were included. The ratio of *i*15∶0 to *a*15∶0 was calculated as a metric that may represent physiological responses to changes in temperature [46]. The fungi-to-bacteria ratio was calculated as (18∶1ω9c+16∶1ω5)/(*a*15∶0+*i*15∶0+15∶0+*i*16∶0+15∶1ω8c +16∶1ω7c+*cy*17∶0+*a*17∶0,+17∶1ω7c). The common fungal biomarker, 18∶2ω6,9, was not a dominant lipid in our samples, as observed for mature lodgepole pine forests in the GYE [41]. Gm+ bacteria lipid markers included: *i*15∶0, *a*15∶0, *i*16∶0, *a*17∶0, *i*17∶0. Gm- lipid markers included: 15∶1ω8, 16∶1ω7, 17∶1ω7, 19∶1ω8t, and 15∶1ω9.

Annual rates of *in situ* net N mineralization were collected using the resin core incubation method [47] and methods are fully are described in Turner et al. [34], who reported annual rates between 2001 and 2004. Here, we incorporated results from the 2002–03 year of incubation when microbial samples were collected. As an independent assessment of soil N cycling dynamics, rates of NH_4_^+^ and NO_3_^−^ transformations were quantified using ^15^N isotope dilution [48], [49] at two of the plots (crown fire plot at Glade and Moran, n = 81/plot), following the methodology described in Smithwick et al. [41]. Aboveground cover (detrital and abiotic: % rock, %unburned litter, % charred litter, % mineral soil, % coarse woody debris, and live plant: % *Lupinus*, % *Ceanothus*, % forbs, % graminoids, % shrubs) was recorded in a 0.25-m^2^ circular sampling frame centered on each core, and soil samples were collected at each sample point for pH (see methods in Turner et al. [34]).

### Statistics

To explore the spatial structure of independent soil and cover characteristics, microbial communities, and N cycling rates, a fully probabilistic spatial process model [35], [50] was used. The model assumes that, conditional on a Gaussian underlying process *S*(*u*), the observed variables *Y*(*u*) are independent and exponentially distributed (evaluated using covariates). The model and observed data were assimilated in a Bayesian framework and posterior distribution was computed from a predefined grid of correlation parameters (*φ and σ^2^*) and relative nugget (*τ^2^.rel*). In this study, a 100×100 correlation parameter grid was used to obtain 100,000 posterior draws. The model can be expressed in a hierarchical framework as follows: Level 1: *Y*(*u*) = *Xβ*+*S*(*u*); Level 2: *S*(*u*) ∼ normal(*0*, *σ* ^2^ *R*(*h*; *φ*)); Level 3: prior(*β*, *σ* ^2^, *φ*). The first level describes a spatial linear trend (*β* = trend parameter) for coordinates (X) or other available spatial covariate data; the second level describes a stationary Gaussian spatial process (S(u)) with mean = 0, variance = *σ^2^* and correlation function R(*h*; *φ*), where *φ* is correlation parameter (range of spatial autocorrelation = 3 *φ*) and *h* is lag distance (vector distance between two locations); and the third level specifies the prior for the model parameters. We chose an exponential correlation function: R(*h*; *φ*) = exp(−*h*/*φ*). Flat prior were chosen for *β* and *φ* and a reciprocal prior for *σ* ^2^. The mean and variance of variables were estimated at individual locations from the predictive distribution using the krige.bayes function of geoR library in R version 2.15.0 [52]. Leave-One-Out cross-validation strategy was used for model validation. Please see [35] for further modeling details.

Stepwise selection (forward and backward) using.stepAIC() function in MASS [51] package of R [52] was used to find the best candidate model for predicting net N mineralization using microbial variables, soil properties, and cover variables as predictors.

## Results

PLFA revealed 26 individual lipid biomarkers that could be uniquely identified across the four plots (**Table 1**). The most dominant lipids (based on relative mole fraction) included 15∶1ω8c, 16∶0, 17∶1ω7c, 18∶1ω9c, and 19∶1ω8c. The relative abundance of individual lipids varied significantly across plots. One lipid (15∶1ω8c) was more common in plots that experienced severe-surface burns compared to crown fires, but no other lipid showed consistent patterns at the site-level, e.g., between plots at Glade versus Moran, or between severity classes (crown versus surface). Microbial lipid abundance varied from 205±15.65 nmol at the Glade crown fire plot to 374±25.03 nmol at the Moran crown fire plot (**Table 2**). The fungi to bacteria ratio averaged 0.88 at the two Glade plots and 0.49 at the Moran plots. Both Gm+ and Gm- bacteria were lowest at the Glade crown fire plot, but did not significantly differ across the other three plots. The stress ratio, i15/a15, was lowest at the crown fire plots compared to the surface plots.

**Table 1:** Relative mole percent (mean (±1 SE), n = 81) of dominant, individual lipids at the post-fire crown and severe-surface burn plots, two years following fire.

Lipid	Glade-crown	Moran-crown	Glade-surface	Moran-surface
11∶0	0.72 *(0.04)*	0.93 *(0.08)*	0.72 *(0.15)*	0.46 *(0.05)*
12∶0	2.90 *(0.11)*	2.08 *(0.07)*	3.43 *(0.11)*	2.17 *(0.07)*
14∶0	4.35 *(0.20)*	2.16 *(0.07)*	3.49 *(0.19)*	2.90 *(0.13)*
15∶0	1.21 *(0.09)*	1.04 *(0.05)*	0.97 *(0.05)*	1.07*(0.04)*
15∶0anteiso	3.02 *(0.10)*	2.70 *(0.10)*	2.55 *(0.10)*	3.22 *(0.14)*
15∶0iso	3.13*(0.10)*	2.91 *(0.09)*	2.86 *(0.09)*	3.90 *(0.13)*
15∶1ω8c	NA	6.66 *(0.66)*	19.82 *(0.00)*	16.22 *(1.75)*
15∶1ω9c	7.75 *(0.88)*	3.72 *(0.36)*	8.64 *(0.60)*	5.64 *(0.38)*
16∶0	12.15 *(0.31)*	8.35 *(0.20)*	10.25 *(0.32)*	10.03 *(0.19)*
16∶02OH	1.47 *(0.09)*	2.56 *(0.45)*	1.82 *(0.24)*	1.74 *(0.12)*
16∶0iso	1.61 *(0.06)*	1.37 *(0.05)*	1.45 *(0.05)*	1.68 *(0.06)*
16∶1ω5c	1.35 *(0.07)*	1.04 *(0.04)*	1.25 *(0.06)*	1.29 *(0.04)*
16∶1ω7c	5.43 *(0.35)*	4.92 *(0.31)*	4.73 *(0.21)*	5.12 *(0.23)*
17∶0anteiso	1.65 *(0.07)*	1.80 *(0.07)*	1.46 *(0.06)*	1.79 *(0.07)*
cy17∶0	2.55 *(0.17)*	2.26 *(0.20)*	2.45 *(0.14)*	2.59 *(0.13)*
17∶0iso	0.79 *(0.05)*	0.53 *(0.03)*	0.76 *(0.05)*	0.72 *(0.03)*
17∶1ω8	1.28 *(0.17)*	1.29 *(0.09)*	1.68 *(0.13)*	1.21 *(0.08)*
17∶1ω7c	3.41 *(0.34)*	9.57 *(0.89)*	6.90 *(0.86)*	7.16 *(0.67)*
18∶0	4.11 *(0.16)*	3.00 *(0.14)*	3.13 *(0.15)*	2.47 *(0.07)*
18∶02OH	3.30 *(0.43)*	4.18 *(0.31)*	3.20 *(0.17)*	3.54 *(0.19)*
18∶1ω9c	16.10 *(0.88)*	9.08 *(0.50)*	12.89 *(0.67)*	11.45 *(0.56)*
18∶2ω6c	4.08 *(0.13)*	2.49 *(0.11)*	3.34 *(0.17)*	3.50 *(0.17)*
18∶3ω6c	2.71 *(0.13)*	1.59 *(0.09)*	2.14 *(0.08)*	2.01 *(0.07)*
19∶0	0.25 *(0.02)*	1.82 *(0.17)*	0.17 *(0.02)*	0.27 *(0.03)*
cy19∶0	0.47 *(0.03)*	0.40 *(0.04)*	0.09 *(0.03)*	0.49 *(0.04)*
19∶1ω8t	7.57 *(0.54)*	9.08 *(0.55)*	9.84 *(0.80)*	9.48 *(0.52)*

**Table 2:** Microbial characteristics (mean (±1 SE), n = 81) of plots two years following the Moran and Glade crown and severe-surface fires in the GYE. Assignment of lipids to microbial groups is explained in the text.

	Glade-crown	Moran-crown		Glade-surface		Moran-surface
Abundance (nmol)	205.25 *(15.65)*		374.19 *(25.03)*		363.90 *(28.00)*	301.73 *(17.80)*
Fungi/Bacteria	0.93 *(0.06)*		0.43 *(0.03)*		0.83 *(0.06)*	0.55 *(0.03)*
Gm+	0.010 *(0.001)*		0.016 *(0.001)*		0.014 *(0.001)*	0.015 *(0.001)*
Gm-	0.020 *(0.002)*		0.048 *(0.005)*		0.049 *(0.005)*	0.040 *(0.004)*
*i*15/*a*15	1.04 *(0.03)*		1.12 *(0.03)*		NA	1.28 *(0.03)*

Laboratory isotopic rates of gross nitrification were higher at the Moran crown fire plot compared to Glade (0.38±0.09 µg NO_3_^−^ g^−1^ d^−1^ versus 0.06±0.02 µg NO_3_^−^ g^−1^ d^−1^) but rates of NH_4_^+^ and NO_3_^−^ consumption were also higher at the Moran crown fire plot (0.83±0.14 µg NH_4_^+^ g^−1^ d^−1^ and 0.61±0.10 µg NO_3_^−^ g^−1^ d^−1^) versus Glade (0.44±0.07 µg NH_4_^+^ g^−1^ d^−1^ and 0.09±0.02 µg NO_3_^−^ g^−1^ d^−1^). As a result, net NH_4_^+^ and NO_3_^−^ mineralization was lower (*p*<0.013) at Moran (−0.47±0.11 µg NH_4_^+^ g^−1^ d^−1^ and −0.24±0.13 µg NO_3_^−^ g^−1^ d^−1^) versus Glade (−0.24±0.04 µg NH_4_^+^ g^−1^ d^−1^ and −0.03±0.01 µg NO_3_^−^ g^−1^ d^−1^).

The best predictors of *in situ* net N mineralization across all four plots included both microbial and aboveground cover variables (**Table 3**). The fungi-to-bacteria ratio was correlated negatively to *in situ* net N mineralization in all final models (i.e., higher relative bacterial abundance was correlated with higher rates of net mineralization). Similarly, fungal abundance, bacterial abundance, or both, were additionally included in final models. Cover variables included in the models were downed coarse wood and *Lupinus* (Glade crown), forbs (Moran crown), or shrubs (Glade surface). Predictive power was low across all models (adjusted R^2^ ranged from 0.11 to 0.29).

**Table 3:** Best predictors of total net nitrogen mineralization in the post-fire crown and severe-surface burn plots.

Glade-crown	Moran-crown	Glade-surface	Moran-surface
− Coarse Wood (%)***	+ Forbs (%)*	−Shrub (%)**	
− Lupinus**	− Gm^−^.	− Bacteria**	− Bacteria****
+ Fungi**	+ Fungi*	+ Fungi**	
− Fungi/Bacteria***	− Fungi/Bacteria**	− Fungi/Bacteria**	− Fungi/Bacteria**

**Notes:**+and – signs indicate the type of correlation and * represents statistically significant relationship at P = 0 ‘****’, P<0.001 ‘***’, P<0.01 ‘**’, P<0.05 ‘*’. Model with best predictors is selected based on minimum AIC value. G and M refer to Glade and Moran sampling locations respectively. The following variables were used in stepwise regression: Rock (%), Charred Litter (%), Fresh Litter (%), Exposed mineral soil (%), Coarse woody debris (%), *Lupinus* (%), *Ceanothus* (%), Forbs (%), Graminoids (%), Shrubs (%), *Pinus contorta* (%), Non-vegetative cover (%), Vegetative cover (%), pH, Abundance, Fungi, Bacteria, Fungi/Bacteria, Gm+, Gm−.

**Figure 1:**
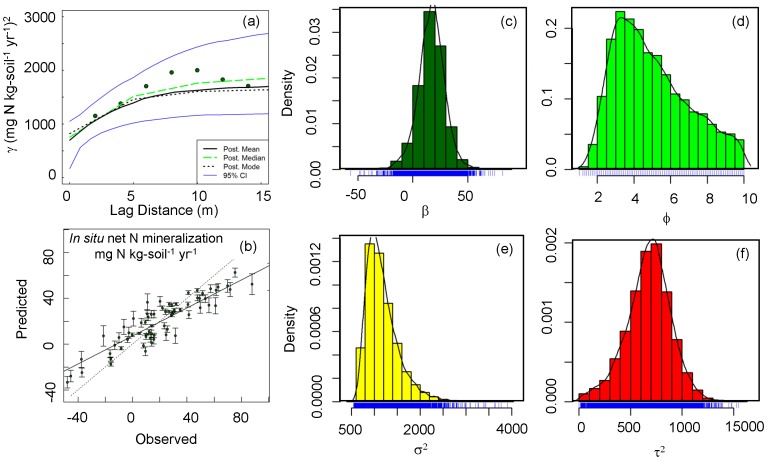
A representative (a) semivariogram, and (b) leave-one-out cross-validation exploring spatial autocorrelation of *in situ* net N mineralization (mg N kg-soil^−1^ yr^−1^), where the dotted line is the 1∶1 line and the solid line is the linear regression fit, and (c–f) posterior distribution of parameters estimated from semivariogram. β is trend, φ is range parameter (range (unit: m)  = 3φ), σ^2^ is sill (unit: (mg N kg-soil^−1^ yr^−1^)^2^), and τ^2^ (unit: (mg N kg-soil^−1^ yr^−1^)^2^) is nugget. Blue lines at the bottom of the histogram indicate the tick marks at the actual data values.

**Figure 2:**
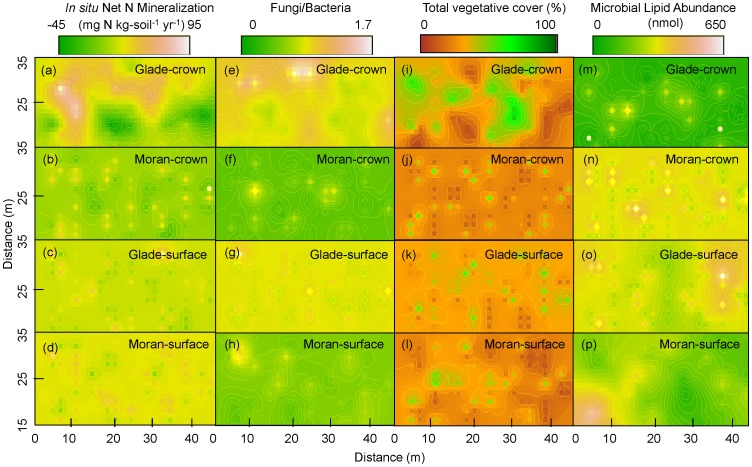
Kriged maps of a–d) *in situ* net nitrogen mineralization rate (mg N kg-soil^−1^ yr^−1^), e–h) Fungi/Bacteria ratio, i–l) total vegetative cover (%), and m–p) microbial lipid abundance (nmol) in the post-fire crown and severe-surface burn plots. Note: discontinuities in mapped patterns reflect locations in which the range was predicted to be less than the minimum sampling distance, or that there was not enough data for continuous spatial prediction.

The ratio of noise to variance (nugget/(nugget+sill)), as well as the cross-validation parameter, indicated strong and significant spatial models given the data (**Table 4** and **Figure 1**). Calculated spatial autocorrelation ranges varied from 1.5 to 13.8 m for microbial lipid abundance, fungi-to-bacteria ratios, *in situ* net N mineralization, total vegetative cover, and total non-vegetative cover (**Table 4**) and R^2^ values were between 0.87 and 0.98. The one exception to this was the calculation of spatial range for microbial lipid abundance in the Moran surface plot, in which the long calculated range (78.9 m) indicates all points were spatially autocorrelated within this plot; notably, variance of the model, though significant, was high (R^2^ = 0.56). The Glade crown fire plot had higher range values across all variables (e.g., 13.8 m for *in situ* N mineralization, compared to an average of 1.9 m across the other 3 plots). Kriged maps of key variables highlight the longer length scales in the Glade crown fire site (**Figure 2**). Patchiness at sub-meter scales across some variables and plots indicates substantial heterogeneity at grains finer than originally sampled (2 m).

**Table 4:** Posterior parameter estimates (mean (5%, 95% CI)) of spatial models in the post-fire crown and severe-surface burn plots. Live plant cover includes % Lupinus, % Ceonothus, % forbs, % graminoids, % shrubs.

	Trend	Range (m)		Sill	Nugget	Nugget/(Nugget+Sill)	R^2^
**Microbial Lipid Abundance (nmol)**							
Glade-crown	204.3 (99.8, 272.9)	4.5 (0.0, 132.9)		10864 (7098, 20234)	7672.2 (3137.4, 15153.3)	0.79 (0.24, 0.99)	0.94
Moran-crown	373.3 (314.8, 423.4)	1.5 (0.0, 100.5)		29362 (20854, 50591)	19549.9 (6444.1, 39124.7)	0.73 (0.16, 0.98)	0.98
Glade-surface	363.5 (156.6, 574.3)	3.0 (0.0, 142.2)		39545 (24616, 71824)	27732.2 (10019.0, 53868.1)	0.80 (0.23, 0.99)	0.93
Moran-surface	313.5 (145.8, 495.4)	78.9 (7.8, 145.2)		20413 (13262, 35273)	16429.6 (10235.0, 22418.8)	0.83 (0.45, 0.99)	0.56
**Fungi-to-Bacteria Ratio**							
Glade-crown	0.9 (0.7, 1.2)	10.5 (1.8, 27.6)		0.14 (0.10, 0.22)	0.10 (0.05, 0.15)	0.78 (0.28, 0.99)	0.89
Moran-crown	0.4 (0.4, 0.5)	3.6 (0.3, 9.6)		0.03 (0.02, 0.05)	0.02 (0.01, 0.03)	0.72 (0.17, 0.98)	0.97
Glade-surface	0.8 (0.7, 0.9)	1.5 (0.0, 6.0)		0.14 (0.10, 0.22)	0.09 (0.03, 0.14)	0.72 (0.16, 0.98)	0.98
Moran-surface	0.5 (0.5, 0.6)	5.1 (1.2, 21.6)		0.03 (0.02, 0.05)	0.02 (0.01, 0.03)	0.75 (0.19, 0.98)	0.94
***In situ*** ** net N mineralization (mg N kg-soil^−1^ yr^−1^)**							
Glade-crown	17.1 (-3.7, 36.1)		13.8 (6.9, 27.0)	1101 (750, 1869)	686.2 (286.1, 1018.0)	0.65 (0.18, 0.97)	0.87
Moran-crown	4.5 (-4.5, 13.5)		2.4 (0.0, 13.8)	792 (562, 1254)	540.6 (190.8, 832.7)	0.74 (0.17, 0.98)	0.97
Glade-surface	17.1 (10.7, 23.8)		1.5 (0.0, 10.5)	558 (402, 866)	379.8 (129.1, 573.7)	0.73 (0.16, 0.98)	0.98
Moran-surface	22.1 (16.8, 27.2)		1.8 (0.0, 6.0)	358 (257, 561)	240.4 (80.6, 354.4)	0.72 (0.16, 0.98)	0.98
**Total live plant cover (%)**							
Glade-crown	29.5 (17.5, 13.3)		9.9 (4.2, 26.1)	413 (290, 669)	282.38 (117.16, 419.5)	0.73 (0.21, 0.98)	0.89
Moran-crown	23.6 (19.3, 28.0)		1.5 (0.0, 4.8)	285 (206, 443)	190.96 (62.18, 277.32)	0.72 (0.15, 0.98)	0.98
Glade-surface	30.2 (25.9, 34.3)		1.8 (0.0, 9.9)	226 (163.0, 351)	153.97 (53.27, 232.56)	0.74 (0.17, 0.98)	0.98
Moran-surface	24.4 (17.5, 30.2)		4.2 (0.3, 23.1)	273 (196, 422)	195.02 (80.41, 299.53)	0.77 (0.22, 0.99)	0.95
**Total detrital+abiotic cover (%)**							
Glade-crown	70.4 (60.5, 82.1)		9.9 (4.2, 26.1)	413 (290, 669)	282.4 (117.1, 419.5)	0.73 (0.21, 0.98)	0.89
Moran-crown	76.3 (71.9, 80.6)		1.5 (0.0, 4.8)	285 (206, 443)	191.0 (62.2, 277.3)	0.72 (0.15, 0.98)	0.98
Glade-surface	69.8 (65.6, 74.0)		1.8 (0.0, 9.9)	226 (163, 351)	154.0 (53.3, 232.6)	0.74 (0.17, 0.98)	0.98
Moran-surface	75.5 (69.5, 82.2)		4.2 (0.3, 23.1)	273 (196, 422)	195.0 (80.4, 299.5)	0.77 (0.22, 0.99)	0.95

Detrital and abiotic cover includes % rock, charred litter, fresh litter, coarse woody debris, and mineral soil.

Note: Adjusted cross-validation R^2^ is reported. Units of nugget and sill are squared units of the corresponding variables.

## Discussion

Laboratory isotopic pool dilution at the crown fire plots confirmed the general patterns of *in situ* N mineralization reported by Turner et al. [37]. Although isotopic pool dilution was not performed at the surface fire plots (because of cost constraints), consistent differences across both approaches at the crown fire sites is reassuring. In general, both resin-core methods and laboratory isotopic pool dilution indicated that nitrification rates were higher at the Moran crown fire site in year 2 compared to the Glade crown fire plot. Higher nitrification at Moran is supported by evidence of larger substrate (NH_4_^+^) pool size for the nitrifier community, and higher pH, both of which are commonly invoked as mechanisms that induce nitrification following fire [17], [53]. Moreover, high rates of nitrification two years following fire is consistent with expected patterns of N cycling following severe fire reviewed by Smithwick et al. [10]. Greater rates of microbial immobilization of NH_4_^+^ revealed by isotopic pool dilution elucidate the potential reason that resin-core net N mineralization rates were lowest at this site. Observed higher levels of microbial lipid abundance and lower fungi-to-bacteria ratios may explain the higher levels of microbial activity.

The inclusion of microbial lipid data into predictive models of net N mineralization two years following severe fire (**Table 3**) supports earlier work showing the importance of fungal and bacterial composition and abundance for explaining post-fire nutrient cycling [17], [54]. In this study, the fungi-to-bacteria ratio was negatively related to net N mineralization rates, which may simply indicate the importance of total bacterial abundance [53] in governing N cycling activity, although differences in C:N between fungi and bacteria and their relative dominance cannot be discounted [55]. Other factors such as pH and charcoal are generally considered important for influencing post-fire N cycling [41], [56], [57]. Soil pH ranged from 4.58 to 5.44 (average 4.9±0.2 (±1 SE)), not significantly different than that in mature stands in the GYE (5.1, [41]) indicating that fire resulted in few differences in soil pH at the plot level two years following fire. Charcoal was not directly assessed in this study, although charred litter was measured and has previously shown to be important in influencing N cycling in the GYE [18], [34]. However, neither pH nor charred litter was selected in the final models.

Aboveground cover was shown previously by Turner et al. [34] to influence net N mineralization rates and pools at individual sampling locations, although the strength of these relationships differed across years and among variables. Here we showed that at the plot-level, *Lupinus* or *Ceanothus*, shrubs, forbs, and the percent of coarse wood influence net N mineralization when microbial variables are included in the analysis. This supports the conclusions by Turner et al. [34] and others [45], [58], [59] that the recovery of biotic and abiotic structure following fire may influence post-fire soil N pools and rates. More specifically, aboveground cover variables appear to be useful proxies of site conditions, such as burn severity [45]. Cover conditions are easily measured and applied in field and modeling approaches, especially those that require intensive spatial sampling designs as required here. Yet it is important to recognize that microbial transformations of soil nutrients are likely mediated by other, more mechanistic variables that are only indirectly correlated with aboveground cover.

Spatial structure of microbial communities and net N mineralization was found to be significant two years following fire. Using semi-variograms, annual net N mineralization was not found to be spatially structured during the 2002–3 incubation year by Turner et al. [34]. However, by using a probabilistic spatial model and cross-validation statistics, we were able to uncover spatial dependence that ranged from 1.5 to 13.8 m across plots (**Table 4** **;** **Figure 2**). Turner et al. also reported spatial dependence for individual biotic and abiotic cover variables that varied from 2.0 to 22.9 m across variables and plots in 2002. Similarly, in this study, the 5% and 95% quantile-based credible interval predicted ranges of total vegetative cover to be between 0 and 26.1 m (**Table 4**), which bounds the earlier results. On average, the predicted range was comparable (1.5 to 9.9 m) to that found for cover variables in Turner et al. in 2003 (1.6 to 4.5 m).

Congruence in aboveground and belowground patterns, especially those relating microbial characteristics to the nutrient cycling processes they mediate, is a long-standing and key priority for unraveling complex ecosystem dynamics [26], [31], [60], [61]. Results here indicated substantial congruence in the scale of patchiness among key variables in both crown and severe surface fire plots two years following fire. Range scales were generally <5 m indicating fine-scale spatial structuring in these post-fire environments. Notably, the Glade crown fire plot appeared to have broader patterns (bigger patches, ∼10–14 m) following fire, but these range scales were also consistent across mineralization, cover, and microbial variables. The fact that spatial structure was similar among ecological variables is suggestive of coupling between pattern (cover, microbial communities) and process (nitrogen mineralization) in these burned plots. We caution however that mean responses hide complex patterns among individual variables and through time [34].

In previous studies, fine-scale patterns of microbial community organization have varied at scales less than 15 cm [62] to scales comparable to those observed in this study (∼5 m, [63]). Moreover, several studies have observed structural variance at multiple scales [62], [64], [65], suggesting that factors affecting microbial community structure and activity (environmental factors, biotic cover, and topographical features) may operate conjointly at several nested scales. Similarly, soil N dynamics have been observed at scales as fine as 2 to 4 mm [66]. In grazed and ungrazed grassland soils, variability of N availability was observed <0.4 m [67]. In an even-aged Norway spruce (*Picea abies*) forest, spatial dependence of N mineralization was 60–95% [65]. The range of variability in soil nitrification has been found to be between 74 cm under wheat cultivation and 10 cm under poplar forests [68]. As with microbial patterning, spatial dependence of nutrient availability has been attributed to patterns at broader scales also, including the spacing of individual trees [68], litter quality [69], and topography or soil type [70].

In this study, the probabilistic spatial models also resulted in calculation of range distances that were less than the 2 m grain of the sampling design, indicating substantial spatial structure at very fine scales. Finer-scale patterns, for example enzymatic activity [71], are likely critical for inferring mechanisms governing microbial and nitrogen dynamics following fire. Model results indicate some locations in which the predicted range was less than the minimum sampling distance (discontinuities or ‘hot spots’ in otherwise continuous gradients) that are suggestive of these finer scale processes and/or the lack of data for continuous spatial prediction. Exploring these patterns would complement the increasing understanding of fire as a modifier of microbial communities and abundance through both direct (e.g., soil heating and oxidation) and indirect (e.g., microclimate, post-fire vegetation dynamics) mechanisms [17], [72]. At the same time, understanding how these mechanisms scale to the plot-level, e.g., the focus of the current study, is needed so as to increase predictive understanding of fire on ecosystem function.

There are several important caveats that should be noted based on the results of our study. First, sites were selected to represent characteristic post-fire environments in the GYE, but because of the necessity of spatially intensive sampling within plots, our sites do not capture landscape-level variation in post-fire environments in the GYE. Other studies have indicated substantial variation in microbial community composition across landscape gradients such as tree species composition [73]. Moreover, our study was restricted to a single year and we are unable to make inferences about annual or seasonal changes in microbial community composition or abundance through time following fire. Chronosequence studies in the GYE that include microbial community composition [41] indicate that microbial communities shift with stage age and ecological succession. In all these microbial studies, consideration of the choice of lipids used for characterizing the microbial community must be weighed carefully, as signature lipids for microbial biomarkers are known to be inconclusive and to vary among methods. In this study we present individual lipid signatures used in the analysis and characterize the microbial community with several metrics, but future efforts should consider alternative approaches (e.g., genetic), and alternative metrics for comparison. In addition, we did not include analysis of spatial patterns of other nutrients (e.g., P, Ca) or nitrogen cycling pathways (e.g., organic N) that likely influence spatial patterns of mineralization, N pools, and microbial communities, largely due to the analytical costs of the spatially intensive sampling design. Together with an absence of understanding of finer-scale mechanisms, exclusion of these variables may explain the relatively low amount of explained variance in the predictive models of net N mineralization at the plot-level. Unraveling these complex and multi-scalar spatial patterns remains challenging.

### Conclusions

Spatial heterogeneity is a critical factor for understanding behavior of macroorganisms and the structure of ecosystems [74]. Understanding complex spatio-temporal interactions in soil is often required to explain and predict these ecosystem processes [75], [76], yet little is known about the scales at which microorganisms are structured [19], [31]. Understanding how microorganisms self-organize in response to disturbance and how this organization affects biogeochemical reactions [77] may help elucidate the mechanisms governing post-disturbance function. In this study, we observed congruent spatial structures (i.e., patchiness) of microbial, cover, and N mineralization variables, suggestive of strong linkages between pattern (microbial abundance, aboveground cover) and process (N mineralization) in burned plots. Moreover, despite low explained variance, microbial variables such as the ratio of fungal to bacterial lipid biomarkers resulted in significant predictive models of *in situ* net N mineralization. However, there was also evidence that spatial patterns were multi-scalar (varying at scales below the minimum sampling distance as well as between 2 and 10 m) and complex among fire plots and variables. This study highlights the potential for developing spatially predictive models of post-fire recovery. Particularly interesting might be studies that compare changes in spatial structure prior to and following a large-scale disturbance event, which may allow for a deeper understanding of the role of disturbance in structuring complex post-fire succession. This may be especially important in heterogeneous landscapes such as those created by fire in the Greater Yellowstone Ecosystem which is expected to experience dramatic changes in fire severity and frequency in coming decades.
